# Neuroimmune related pathway may involve in neuropathic pain after brachial plexus injury: a clinical and experimental discovery

**DOI:** 10.1515/biol-2026-2001

**Published:** 2026-05-11

**Authors:** Zhehui Tu, Bengang Qin, Liqiang Gu

**Affiliations:** Department of Pediatric Orthopedics, Guangzhou Women and Children’s Medical Center, Guangzhou Medical University, Guangzhou, Guangdong, 510623, China; Department of Microsurgery, Trauma and Hand Surgery, The First Affiliated Hospital of Sun Yat-Sen University, Guangzhou, 510080, China

**Keywords:** microarray, bioinformatics analysis, brachial plexus avulsion injury, neuropathic pain, signal pathway, peripheral nerve injury

## Abstract

To find the pathways of neuropathic pain after brachial plexus avulsion injury, we screen out key molecules or related signal pathways. Serum samples were collected from 20 patients with brachial plexus injury (BPI) and 10 healthy controls. A BPI rat model was constructed, divided into control, sham, and operated groups. Subtype injuries (upper, lower, complete avulsion) were further modeled. Protein profiles were analyzed using Raybiotech GSH-INF-3 and AAH-NEU-2 antibody microarrays. In clinical samples, 5 cytokines (MMP-3, CNTF, GM-CSF, IL-18, TGF-β) were differentially expressed between BPI patients and healthy controls, among which IL-18, CRP, and GM-CSF were elevated in the pain group compared to the painless group. In rats, serum cytokines showed no significant differences; however, nerve tissue analysis revealed increased levels of IL-6, IL-13, MCP-1, and TNF-α in the operated BPI group compared to the sham group. Histological and immunohistochemical analyses showed progressively severe nerve degeneration and inflammatory responses in upper, lower, and complete injury models. There were signal pathways related to autoimmune diseases screened out, such as IL-17 signaling pathway, inflammatory bowel disease, and Th1 and Th2 cell differentiation. This study suggests that cytokines may affect neuropathic pain in inflammatory pathway and neuroimmune pathway.

## 1 Introduction

Brachial plexus injury (BPI) represents a severe form of peripheral nerve damage, frequently resulting in long-term upper limb dysfunction and disability in both adults and children [1]. BPI is associated with a high rate of disability and commonly causes sensory disturbances, muscle paralysis, or atrophy. Ultimately, it leads to partial or complete loss of motor and sensory function in the upper limbs. These impairments impose a significant burden on patients’ daily activities and mental health [2], 3]. Approximately 30–90 % of patients with BPI suffer from neuropathic pain, which is often severe [4]. This pain is characterized by a persistent burning sensation accompanied by intermittent paroxysmal pain, primarily involving the forearm and hand [5], 6]. However, the underlying mechanisms of NPBPI remain poorly understood, which restricts the effectiveness of current treatment strategies.

One widely accepted theory posits that cascade inflammatory reactions of peripheral immune cells following nerve injury, the recruitment of inflammatory cells, the expression levels of cytokines, and the intensity of the immune response are positively related to the severity of neuropathic pain [7], 8]. When a peripheral nerve is injured, it triggers the release of signaling molecules that activate microglia in the central nervous system, leading to the production of pro-inflammatory mediators, such as IL-1β, TNF-α, and IL-6, which are crucial for the onset and persistence of neuropathic pain [9]. Moreover, recent studies in rats with brachial plexus avulsion have demonstrated that elevated EZH2 levels in anterior cingulate cortex microglia exacerbate neuropathic pain by suppressing autophagy [10]. The indirect mechanisms of BPI resulting from radiation exposure are closely linked to the inflammatory response and the production of cytokines [11]. Importantly, therapeutic interventions targeting inflammatory pathways may show great promise. Edaravone may partially enhance motor neuron survival and promote motor function recovery by suppressing pyroptosis and neuroinflammation, accompanied by decreased levels of IL-1β, IL-6, TNF-α, IL-18, p-p65, NLRP3, and GSDMD [12]. These findings highlight the pivotal role of inflammation in the pathophysiology of neuropathic pain following BPI.

A recent microarray analysis has pinpointed PIK3CB, HRAS, and JUN as potential biomarkers of brachial plexus avulsion-induced neuropathic pain in a rat model of male BPI [13]. British experts used serum samples from 12 patients and validated, in a rat model that TIMP1 could be a related marker for chronic lower back pain [14]. Up to now, no studies have employed microarray techniques to analyze serum samples from patients with BPI. Therefore, in this study, serum samples were collected from both BPI patients and SD rats to identify specific inflammatory factors and signaling pathways potentially associated with neuropathic pain following BPI.

## 2 Methods

Serum samples were collected from patients with chronic pain subsequent to BPI. Concurrently, a BPI animal model was established in SD rats. These samples, along with relevant public datasets, were used to identify differentially expressed factors and proteins linked to neuropathic pain.

### 2.1 Patients

A total of 20 patients diagnosed with BPI were recruited in this study. All patients were admitted to the Department of Microsurgery at the First Affiliated Hospital of Sun Yat-sen University between November 2017 and February 2018, and had completed the “Brachial Plexus Injury Survey Questionnaire”. The patient group consisted of 15 individuals with neuropathic pain (VAS ≥ 1) and 5 individuals without pain symptoms (VAS = 0). The cohort included patients with complete, upper trunk, and lower trunk brachial plexus injuries. The group comprised 16 males and 4 females, aged 6–68 years (mean age: 37.1 years). Additionally, 10 healthy volunteers (5 males and 5 females) were recruited as controls, with an age range of 22–36 years (mean age: 26.7 years). All control participants had no neurological or systemic inflammatory conditions. The inclusion criteria for the BPI group were as follows: (1) a confirmed diagnosis of BPI; and (2) completion of the neuropathic pain survey. The exclusion criteria for both groups included: (1) comorbidities that could affect pain perception (such as diabetes, cancer, or chronic pain syndromes); (2) neurological or psychiatric disorders, including major depression or substance abuse history; (3) preschool – aged children; (4) inability to comprehend or complete the questionnaire; and (5) poor compliance or inability to attend postoperative follow-up. Peripheral venous blood (3 mL) was collected from all participants. Blood from healthy volunteers was drawn after an overnight fasting.

The supernatant was collected and analyzed using GSH-INF-3 and AAH-NEU-2 antibody microarray kits (RayBiotech). Data normalization and analysis were performed using the proprietary software provided by RayBiotech.

**Informed consent:** Informed consent has been obtained from all individuals included in this study.

**Ethical approval:** The research related to human use has been complied with all the relevant national regulations, institutional policies and in accordance with the tenets of the Helsinki Declaration, and has been approved by the Clinical Research and Experimental Animal Ethics Committee of the First Affiliated hospital of SYSU (Approval No. [2017] 291).

#### 2.1.1 Animal experiment

Thirty specific pathogen free (SPF)-grade male SD rats (200–260 g) were provided by the Guangdong Provincial Medical Laboratory Animal Center (License No. SCXK (Yue) 2013-0002) and housed at the Experimental Animal Center of Sun Yat-sen University. All rats were housed under clean-grade conditions, with 2–3 animals per cage, and were provided with *ad libitum* access to food and water. 30 SPF experimental SD rats were randomly assigned to three groups, with 10 rats in each group, including the blank control group, the operated group, and the sham-operated group. All animals were housed and maintained by staff at the Experimental Animal Center of Sun Yat-sen University.

**Ethical approval:** The research related to animal use has been complied with all the relevant national regulations and institutional policies for the care and use of animals, and has been approved by the Experimental Animal Ethics Committee of Zhongshan School of Medicine, Sun Yat-sen University (Approval No. 2017-256).

#### 2.1.2 Surgery procedures

**Figure 1:**
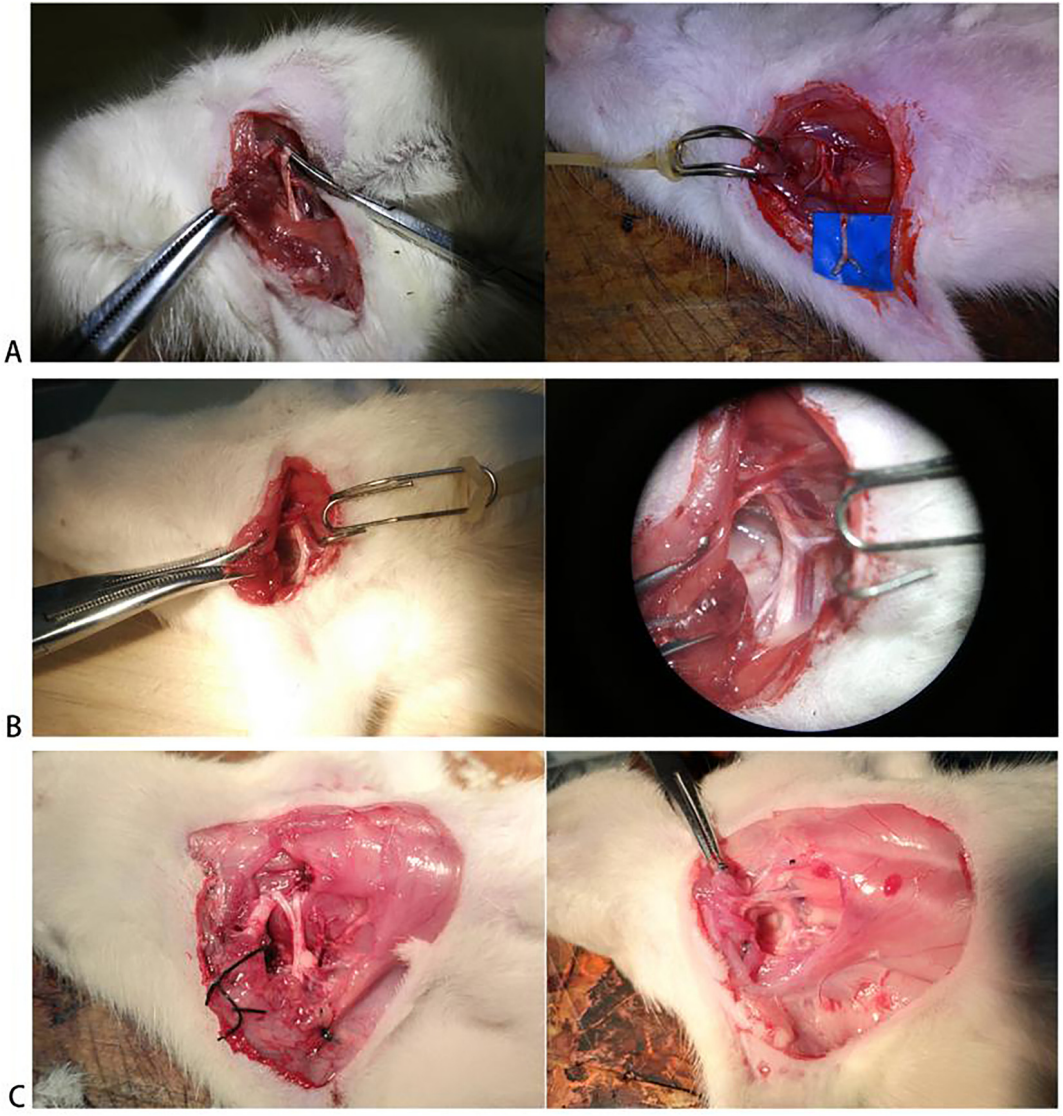
SD rats surgery. A: Lower brachial plexus avulsion injury model; B: Sham-operated group; C: 7 days post-operation (left) and 14 days post-operation (right).

After intraperitoneal anesthesia, the rats were secured in the supine position. Following disinfection, a 0.5 % bupivacaine solution was administered subcutaneously for local anesthesia. An oblique incision approximately 4 cm in length was made 0.5 cm below and parallel to the right clavicle, extending from the sternum to the upper arm. The pectoralis major muscle was bluntly dissected using vascular forceps, and a self-made retractor was used to pull the muscles on both sides apart. The cords and divisions of the brachial plexus were exposed, and the nerves were carefully dissected away from the subclavian vessels. Micro-mosquito forceps and micro-tweezers were used to sequentially separate the lateral, posterior, and medial cords, followed by the anterior and posterior divisions. The lower trunk of the brachial plexus was then identified and isolated based on the division positions. The distal end of the lower trunk was gently clamped with small vascular forceps, and the lower trunk was avulsed. Finally, the incision was sutured layer by layer and disinfected (Figure 1). To investigate the temporal dynamics of inflammation following brachial plexus injury, surgical trauma was categorized as acute (day 7) or chronic (day 14) based on postoperative time points, corresponding to early and sustained/resolving inflammatory phases, respectively. Blood samples and proximal nerve stump tissues were collected at 7 and 14 days post-operation, respectively, and analyzed using rat-specific inflammatory cytokine antibody microarray kits.

#### 2.1.3 Construction of BPI subtypes

Twenty male SPF-grade rats (180–220 g) were procured from Chengdu Dossy Experimental Animals Co., Ltd. (NO. 511214900011712, China, Chengdu) and initially placed in a quarantine room within an SPF barrier system for a 7-day isolation and observation period. After quarantine, the rats were transferred to standard SPF housing rooms for routine care under a 12-h light/dark cycle at a controlled temperature of 26 °C. Following anesthesia induction via inhalation of 2 % isoflurane, a supraclavicular horizontal incision was made to expose the brachial plexus. The skin was incised, the sternocleidomastoid muscle was transected, and the omohyoid muscle was retracted laterally to expose the brachial plexus situated between the scalene muscles. In the sham group, no further surgical manipulation was performed, and the incision was closed. In the upper trunk injury group, the C5–C6 nerve roots were carefully isolated and pulled out using forceps before closing the incision. In the lower trunk injury group, the C8-T1 roots were isolated and pulled. In the complete brachial plexus avulsion group, all segments from C5 to T1 were isolated and avulsed. Subsequently, the muscle and skin were sutured, and povidone – iodine was applied. Rats were placed on a heating pad until they fully regained consciousness and then returned to their cages for continued housing.

#### 2.1.4 Paw withdrawal latency (PWL) assessment

Thermal nociceptive thresholds were assessed using a plantar test system (Jinan Yiyan Technology Development Co., Ltd., China, Jinan) following the Hargreaves method. All rats were acclimatized in a transparent observation chamber (20 × 20 × 25 cm) positioned on a glass platform for 30 min before testing. A radiant heat source was directed onto the mid-plantar surface of the hind paw. The PWL, defined as the time from thermal stimulus onset to paw withdrawal, was automatically recorded. Measurements were performed at baseline (pre-surgery) and on days 3, 7, 14, and 28 post-surgery. Each hind paw was tested three times at 5-min intervals, and the average value was utilized for analysis. A cut-off time of 20 s was established to prevent tissue damage. All assessments were carried out in a quiet environment by an investigator who was blinded to the experimental groups.

#### 2.1.5 Hematoxylin and eosin (HE) staining

To assess morphological changes in the nerve tissues following BPI, HE staining was conducted. Proximal nerve tissues of the brachial plexus were collected from rats in the sham, upper trunk injury, lower trunk injury, and complete injury groups. The tissues were fixed in 4 % paraformaldehyde for 24 h, embedded in paraffin, and sectioned into 4-µm-thick slices. Subsequently, sections were stained with hematoxylin (C0105S; Beyotime, Shanghai, China) for 5 min and then counterstained with eosin for 30 s. After mounting with neutral gum, the stained sections were examined and photographed using a light microscope (Olympus, Tokyo, Japan).

#### 2.1.6 Immunofluorescence

The expression levels of TrkA, TRPV1, CHAT, and CGRP in the proximal nerve tissues of the brachial plexus was assessed by immunofluorescence staining. Tissue samples were fixed in 4 % paraformaldehyde overnight at 4 °C, dehydrated through a graded series of sucrose solutions, and embedded in an optimal cutting temperature (OCT) compound. Coronal sections (5 μm thick) were prepared using a cryostat (Leica, Wetzlar, Germany) and subjected to immunofluorescence labeling. After blocking with 5 % bovine serum albumin (BSA) for 30 min at 37 °C, sections were incubated overnight at 4 °C with rabbit anti-TrkA antibody (1:500; ab302524, Abcam, Cambridge, USA), rabbit anti-TRPV1 antibody (1:500; ab305299, Abcam), rabbit anti-CHAT (1:500; ab181023, Abcam), and mouse anti-CGRP antibody (1:500; ab81887, Abcam). After three washes with phosphate-buffered saline containing 0.1 % Tween-20 (PBST), the sections were incubated for 1 h at room temperature in the dark with DyLight 488-conjugated goat anti-rabbit IgG (EarthOx, E032220) and DyLight 594-conjugated goat anti-mouse IgG (EarthOx, E032210). Fluorescence images were captured using a Leica LCS SP8 STED confocal microscope.

#### 2.1.7 Immunohistochemical (IHC)

Paraffin-embedded sections (4 μm thick) were deparaffinized in xylene and then rehydrated through a series of graded ethanol solutions. Antigen retrieval was carried out by pressure-cooking the sections in EDTA buffer (pH 8.0; ZSGB-BIO, China) for 25 min. Endogenous peroxidase activity was inhibited using 0.3 % hydrogen peroxide (H_2_O_2_). Subsequently, the sections were blocked with 5 % BSA at room temperature for 30 min and incubated overnight at 4 °C with primary antibodies against S100 (1:200; ab52642, Abcam) and NF200 (1:200; ab8135, Abcam). After thorough washing, sections were incubated with an HRP-conjugated secondary antibody (Abcam) at 37 °C for 60 min in the dark. 3,3′-diaminobenzidine (DAB) was used for chromogenic development, followed by hematoxylin counterstaining and mounting with neutral gum. Finally, the stained sections were examined under a light microscope (Olympus).

#### 2.1.8 Analysis method

The raw microarray data underwent normalization and differentially expressed proteins were identified utilizing analysis software supplied by RayBiotech. Subsequent bioinformatics analyses encompassed principal component analysis (PCA), hierarchical clustering heatmap generation, Gene Ontology (GO) enrichment analysis, and Kyoto Encyclopedia of Genes and Genomes (KEGG) pathway analysis. GO analysis, which serves as an international standard for gene function classification, includes three categories: molecular function (MF), biological process (BP), and cellular component (CC). GO enrichment enables the identification of key biological functions and related genes associated with phenotypic changes. KEGG is a comprehensive database for the systematic analysis of gene functions and genome information, facilitating the construction of gene and expression networks. KEGG pathway enrichment analysis highlights significantly altered pathways, providing insights into the underlying biological regulatory mechanisms under specific experimental conditions.

## 3 Results

Blood samples were obtained from 20 patients with BPI and 10 healthy individuals. Using the VAS, patients with a score ≥1 were categorized into the pain group, while those with a score of 0 were assigned to the painless group. Among the 20 patients, 15 belonged to the pain group and 5 to the painless group. According to injury type, 10 patients had complete BPI, 6 had upper BPI, and 1 had lower BPI.

A total of 29 rat nerve tissue specimens and 29 corresponding serum samples were collected. For each group, 5 samples were obtained on days 0, 7, and 14 post-surgery. Additionally, 1 sample was collected from each sham – operated group, and 3 samples were obtained from the operated groups on day 90.

### 3.1 Clinical sample results

**Figure 2:**
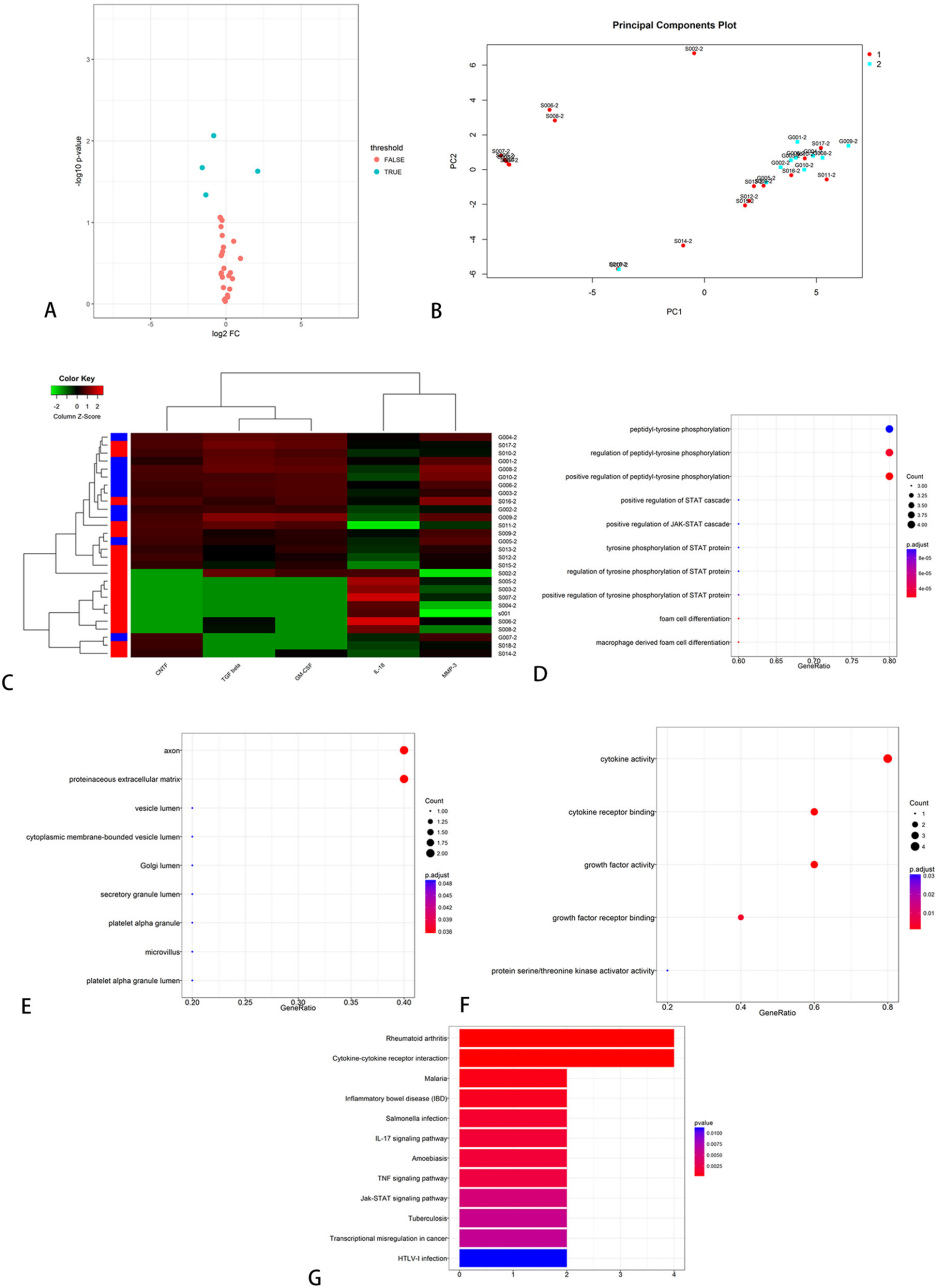
KEGG pathway analysis between brachial plexus injury patients and healthy group.

**Figure 3:**
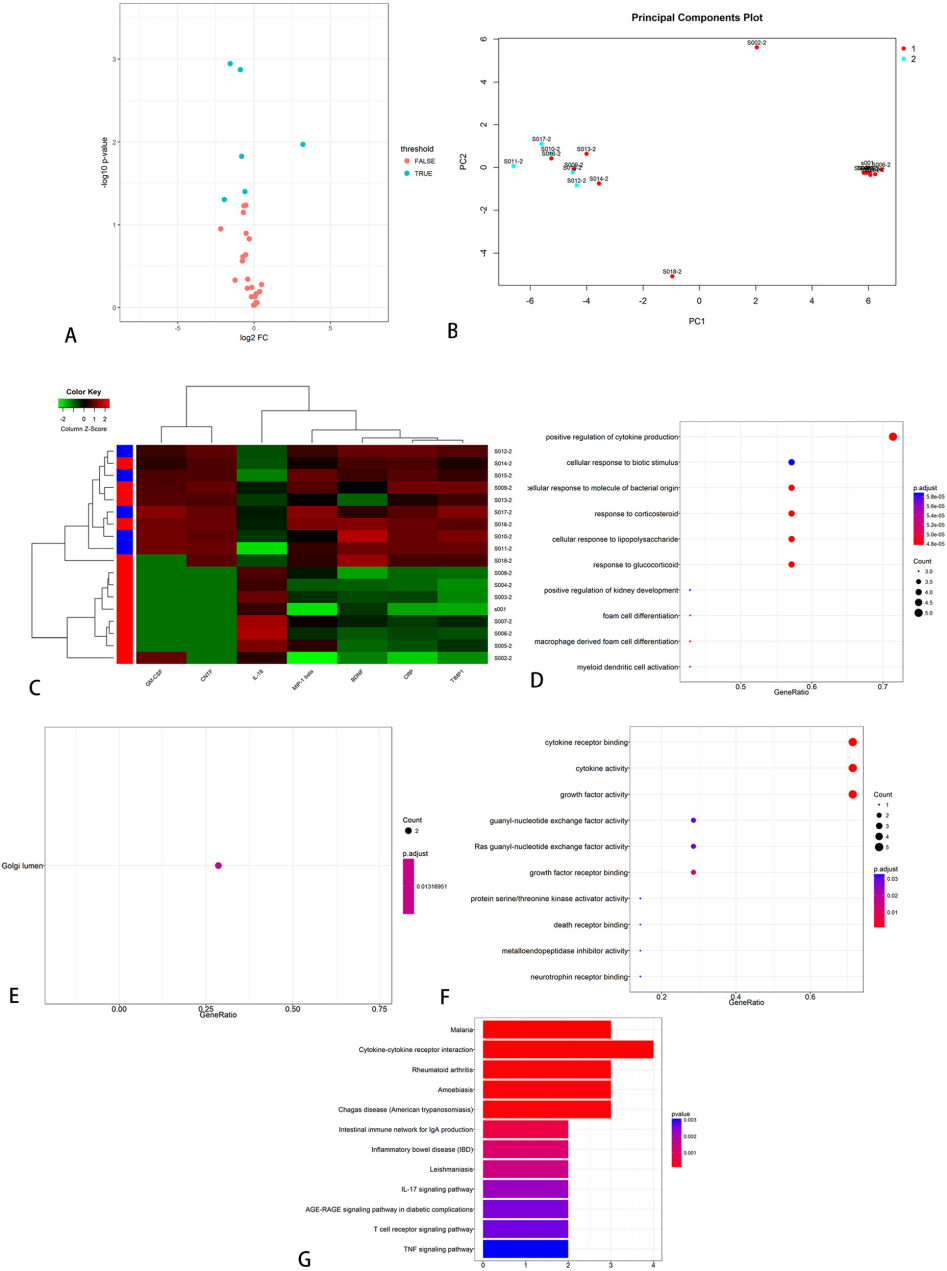
KEGG pathway analysis between pain and painless group in BPI patients. A: GSH-INF-3 kit; B: AAH-NEU-2 kit.

Within the AAH-NEU-2 panel, five factors, including MMP-3, CNTF, GM-CSF, IL-18, and TGF-β, showed differential expression between BPI patients and healthy controls. Notably, IL-18 expression was elevated in the BPI group compared to the healthy control group (Table 1). KEGG enrichment analysis unveiled 12 significantly enriched pathways (Figure 2). A total of 14 proteins demonstrated differential expression between pain and painless patients after brachial plexus avulsion injury. These included 7 factors from the GSH-INF-3 kit (Eotaxin, G-CSF, IL-1ra, IL-4, PDGF-BB, TNF RI, and TNF RII) and 7 from the AAH-NEU-2 kit (CRP, CNTF, TIMP1, IL-18, BDNF, MIP-1, and GM-CSF). The expression levels of all 14 proteins were significantly higher in the pain group compared to the painless group (Table 2). KEGG enrichment analysis pinpointed 17 significantly enriched pathways associated with these differentially expressed proteins (Figure 3). Only one differential protein, MCP-1 (from the GSH-INF-3 kit), was identified between patients with upper and lower BPI, which was inadequate for further analysis.

**Table 1:** Differentially expressed proteins between patients and healthy people after brachial plexus injury.

Factors	Log2FC	P value
MMP-3	−0.76874	0.000139
CNTF	−0.81102	0.008601
GM-CSF	−1.57444	0.021362
IL-18	2.104376	0.023614
TGF beta	−1.3319	0.04599

**Table 2:** Differential expression of inflammatory factor microarray in patients with chronic pain and painless patients after brachial plexus injury.

Factors	log2FC	P value
Eotaxin	0.7446	0.0222
G-CSF	0.5807	0.0210
IL-1ra	1.1984	0.0359
IL-4	0.5335	0.0040
PDGF-BB	0.4165	0.0444
TNF RI	0.4803	0.0076
TNF RII	0.4437	0.0204

### 3.2 SD rats results

#### 3.2.1 Thermal nociceptive sensitivity assessed by hot plate test following different types of BPI

To evaluate thermal pain sensitivity, PWL was determined at baseline and on postoperative days 3, 7, 14, and 28. At baseline, no significant differences were detected among the sham, BPI-upper, BPI-lower, and BPI-avulsion groups. On day 3, PWL significantly decreased in the BPI-lower groups compared to the sham group, indicating the development of hyperalgesia. In contrast, the BPI-avulsion group demonstrated a marked increase in latency, suggestive of sensory block. This trend persisted and intensified on days 7, 14, and 28, with the BPI-avulsion group consistently showing significantly prolonged latencies, while the BPI-upper and BPI-lower groups further reduced latencies compared to sham (Supplementary Figure 1). These findings indicate that partial injuries elicit sustained thermal hypersensitivity, whereas complete root avulsion leads to prolonged thermal hypoalgesia or sensory loss.

#### 3.2.2 Histological and immunohistochemical analysis of BPI subtypes

We further conducted an in – depth assessment of the pathological characteristics and inflammatory responses in different types of BPI. HE staining showed that the sham group had intact nerve structures with orderly arranged fibers. The upper trunk injury group exhibited moderate disorganization and vacuolization of nerve fibers. In the lower trunk injury group, more severe edema, disrupted architecture, and inflammatory cell infiltration were observed. The complete injury group presented with extensive vacuolization, fiber fragmentation, and severe structural disruption (Supplementary Figure 2A). Immunofluorescence staining revealed minimal expression of TrKA and TRPV1 in the sham group, with progressively increased fluorescence in the injury groups, particularly in the complete injury group (Supplementary Figures 2B and C). To assess cholinergic and sensory neurons, we evaluated the expression of choline acetyltransferase (CHAT) and calcitonin gene-related peptide (CGRP). CHAT, a pivotal enzyme in acetylcholine synthesis, serves as a marker for cholinergic neurons, while CGRP was utilized as an indicator of sensory nerve fibers involved in neurogenic inflammation [15], 16]. CHAT expression showed no significant difference between groups, indicating cholinergic neurons were relatively unscathed by BPI. CGRP expression was markedly upregulated in all injury groups, suggesting increased sensory nerve activity (Supplementary Figure 3A). We further explored nerve integrity and regeneration by performing immunohistochemical staining for S100 and NF200. The sham group displayed dense S100-positive Schwann cells and well-aligned NF200-positive axons. The upper trunk injury group showed reduced S100 and attenuated NF200 staining. The lower trunk group exhibited moderate Schwann cell loss and partial axonal fragmentation. Notably, the complete injury group had sparse S100-positive cells and severely disorganized or diminished NF200-positive axons, indicating pronounced nerve degeneration (Supplementary Figure 3B).

#### 3.2.3 Differential expression factor analysis in BPI rats

**Figure 4:**
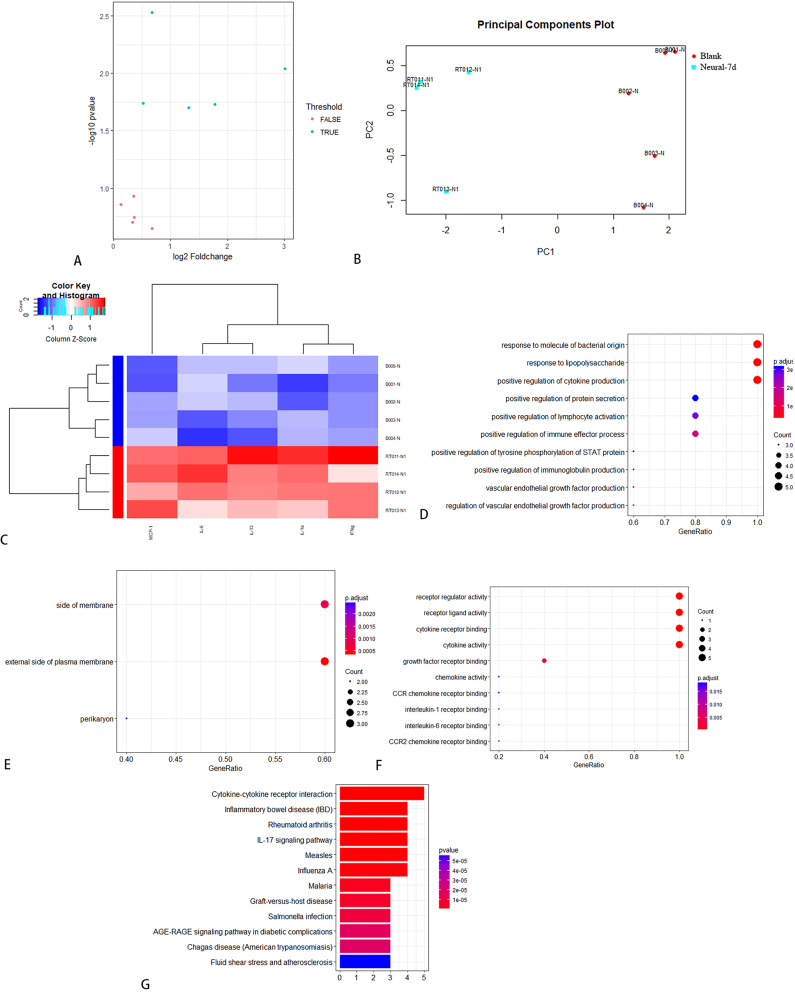
KEGG pathway analysis between operated and control group in SD rats on day 7.

**Figure 5:**
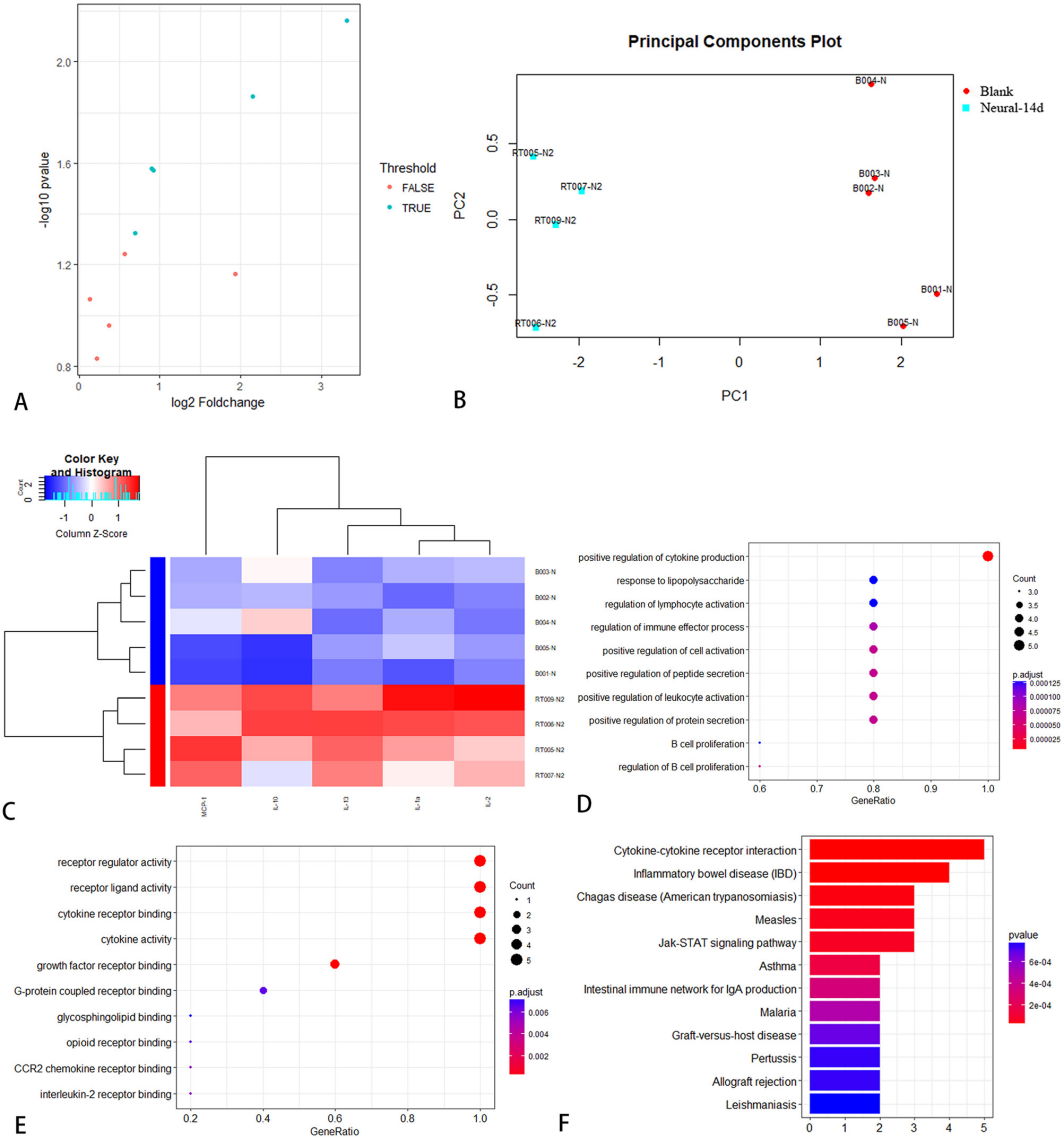
KEGG pathway analysis between operated and control group in SD rats on day 14.

Serum samples from SD rats failed to reveal any statistically significant differentially expressed proteins. From neural tissue samples, five factors (IFN-γ, IL-1α, IL-6, IL-13, and MCP-1) exhibited significantly elevated expression levels in the operated group compared to the control group on day 7 (Table 3). KEGG enrichment analysis identified 35 significantly enriched pathways, among which the top 12 were visually represented in Figure 4. Additionally, IL-6, IL-13, and MCP-1 demonstrated differential expression between the operated and sham-operated groups, suggesting that these cytokines may be associated with surgical trauma. On day 14, five cytokines (IL-1α, IL-2, IL-10, IL-13, and MCP-1) revealed differential expression between the operated and control groups. Notably, the operated group exhibited higher expression levels (Table 4). KEGG enrichment analysis identified 25 significantly enriched pathways, among which the top 12 were visually presented in Figure 5.

**Table 3:** Differentially expressed proteins in nerve tissues of operation group and blank group after 7 days of injury of inferior brachial plexus in SD rats.

Factors	log2FC	P value
IFNg	0.512156	0.018129
IL-1a	0.675703	0.002968
IL-6	1.774066	0.018571
IL-13	1.315474	0.019807
MCP-1	3.015074	0.009127

**Table 4:** Differentially expressed proteins in nerve tissues of operation group and blank group after 14 days of inferior brachial plexus injury in SD rats.

Factors	log2FC	P value
IL-1a	0.918415	0.026769
IL-2	0.903215	0.026442
IL-10	0.693646	0.047302
IL-13	3.320081	0.006883
MCP-1	2.154989	0.013676

**Figure 6:**
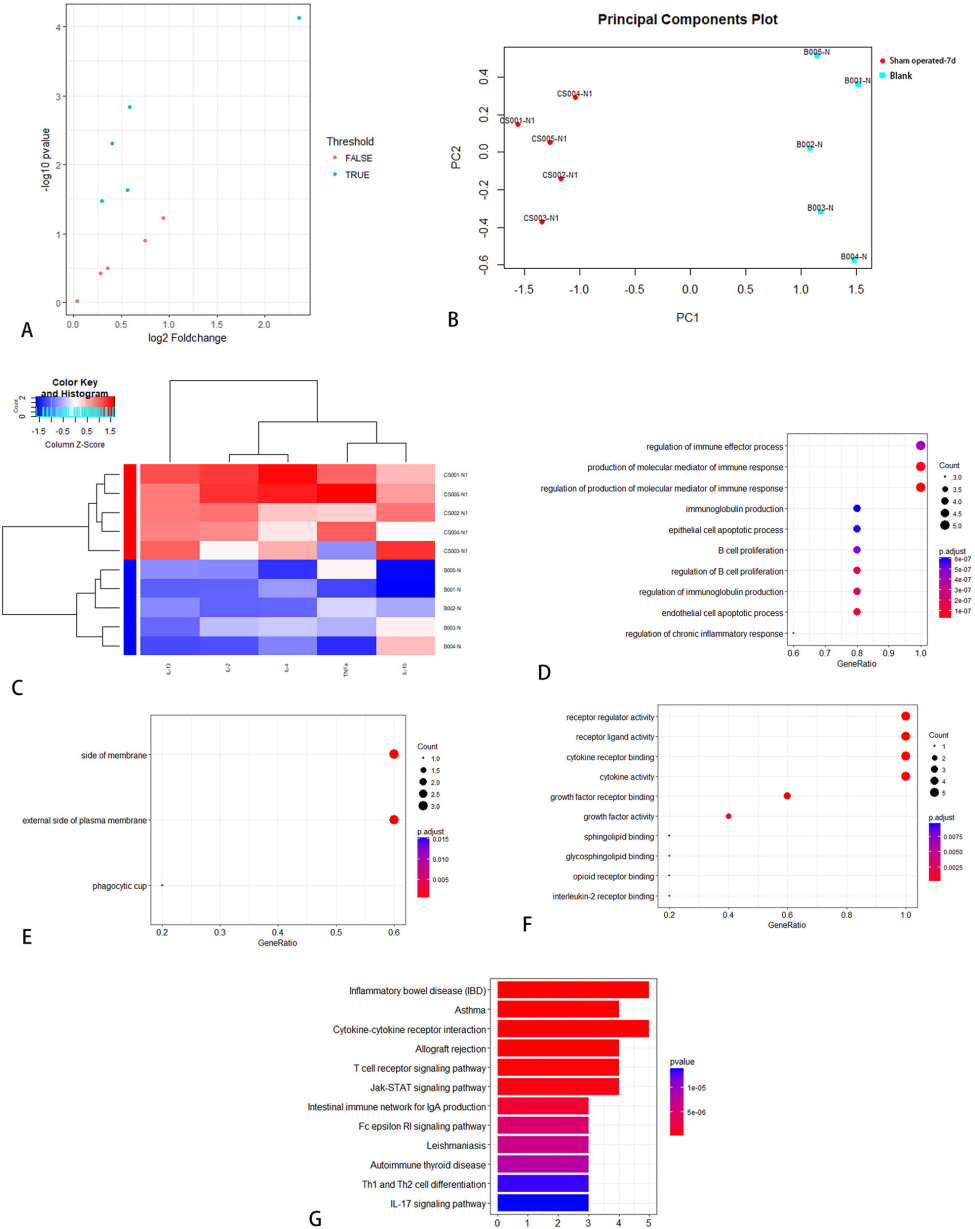
KEGG pathway analysis between sham operation group and blank group in SD rats on day 7.

**Figure 7:**
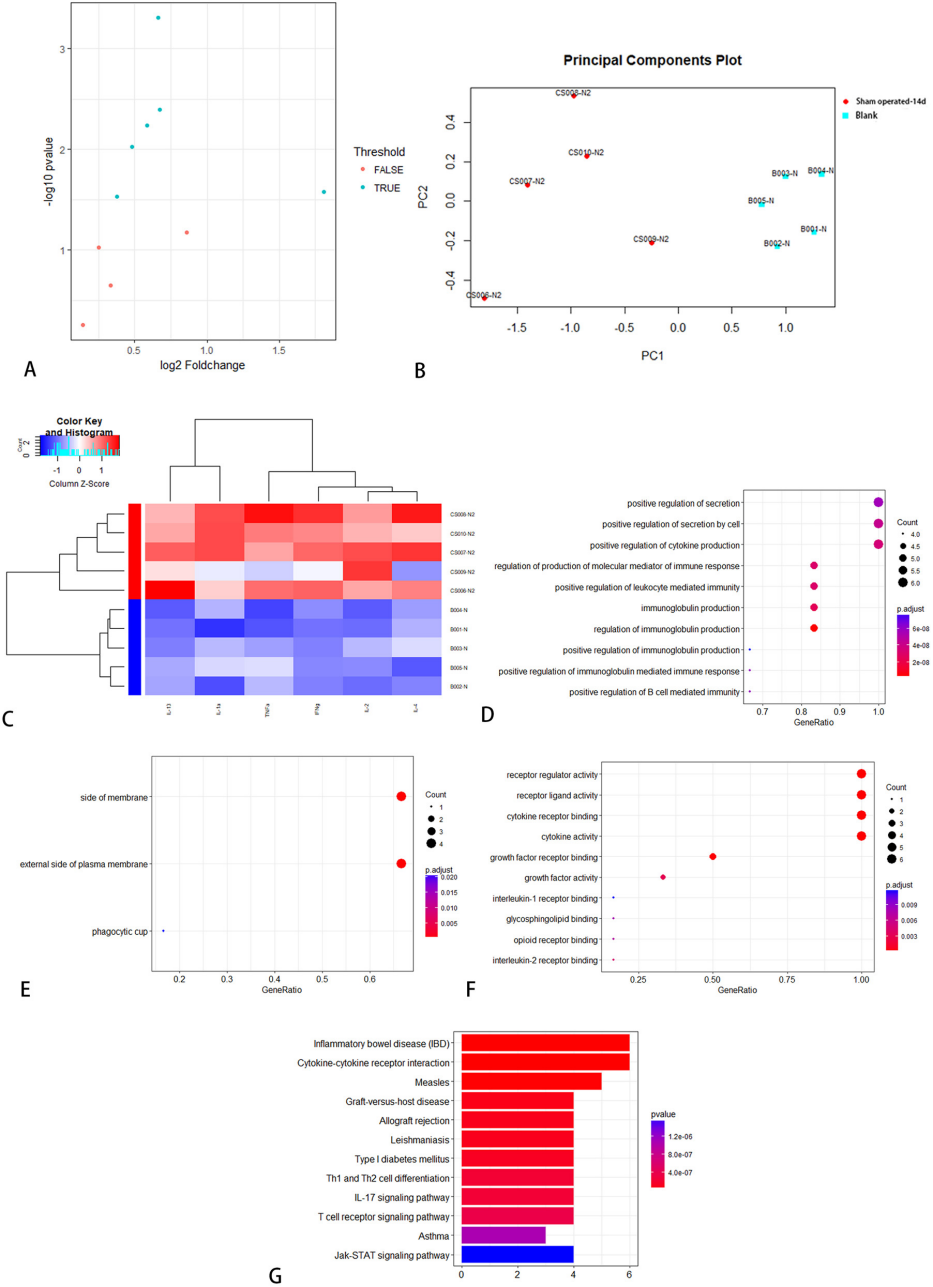
KEGG pathway analysis between sham operation group and blank group in SD rats on day 14.

**Figure 8:**
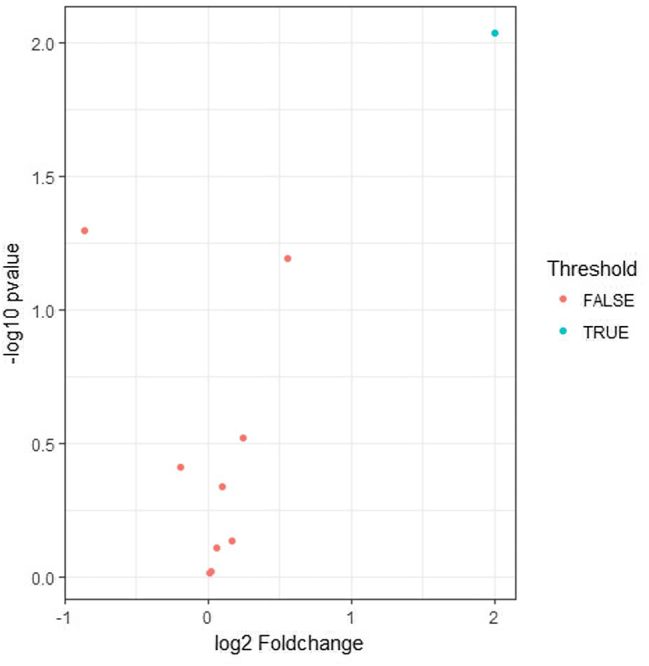
IL-13 was higher on day 14 than day 7 in operated group.

**Figure 9:**
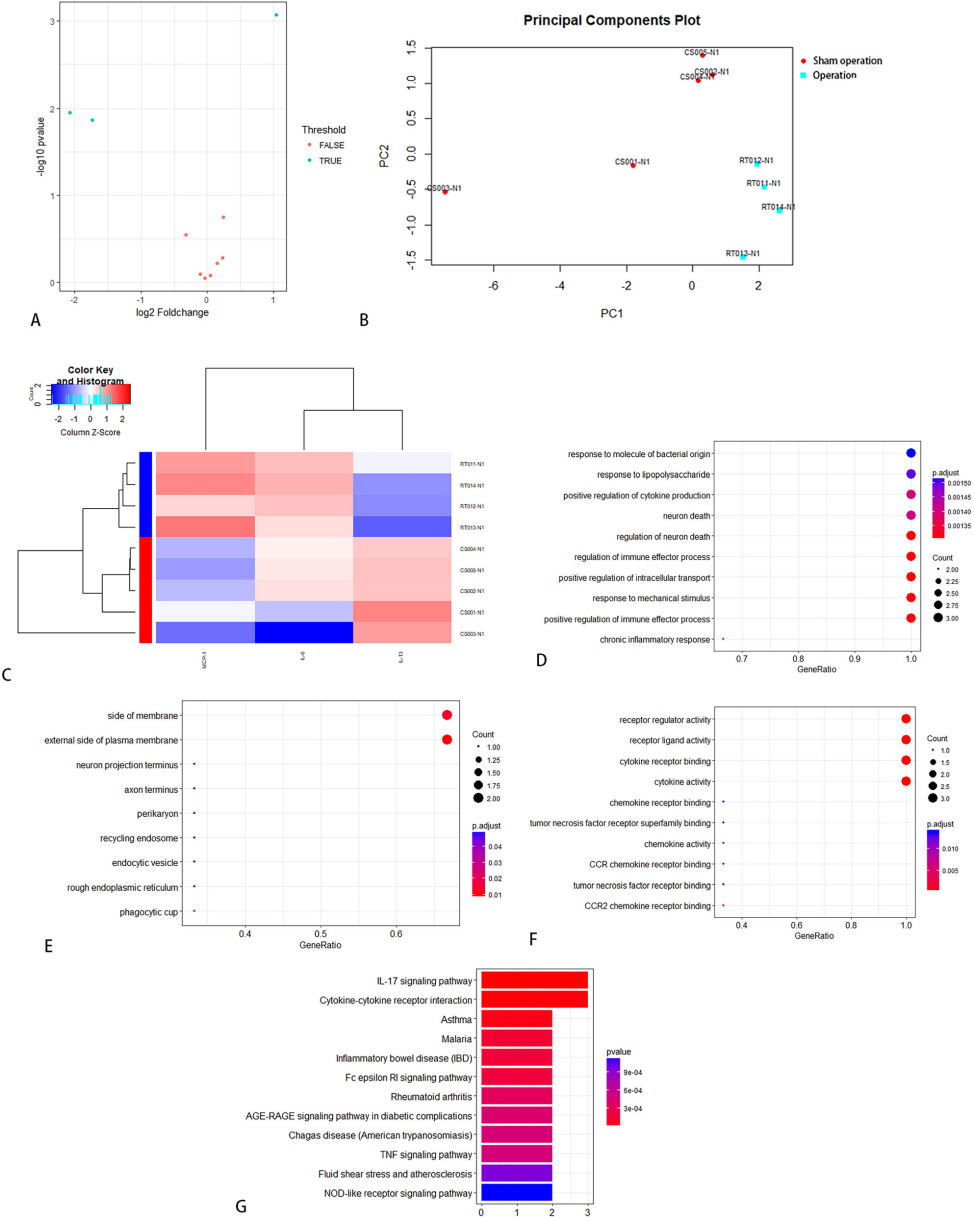
KEGG pathway analysis between operation group and sham operation group in SD rats on day 7.

**Figure 10:**
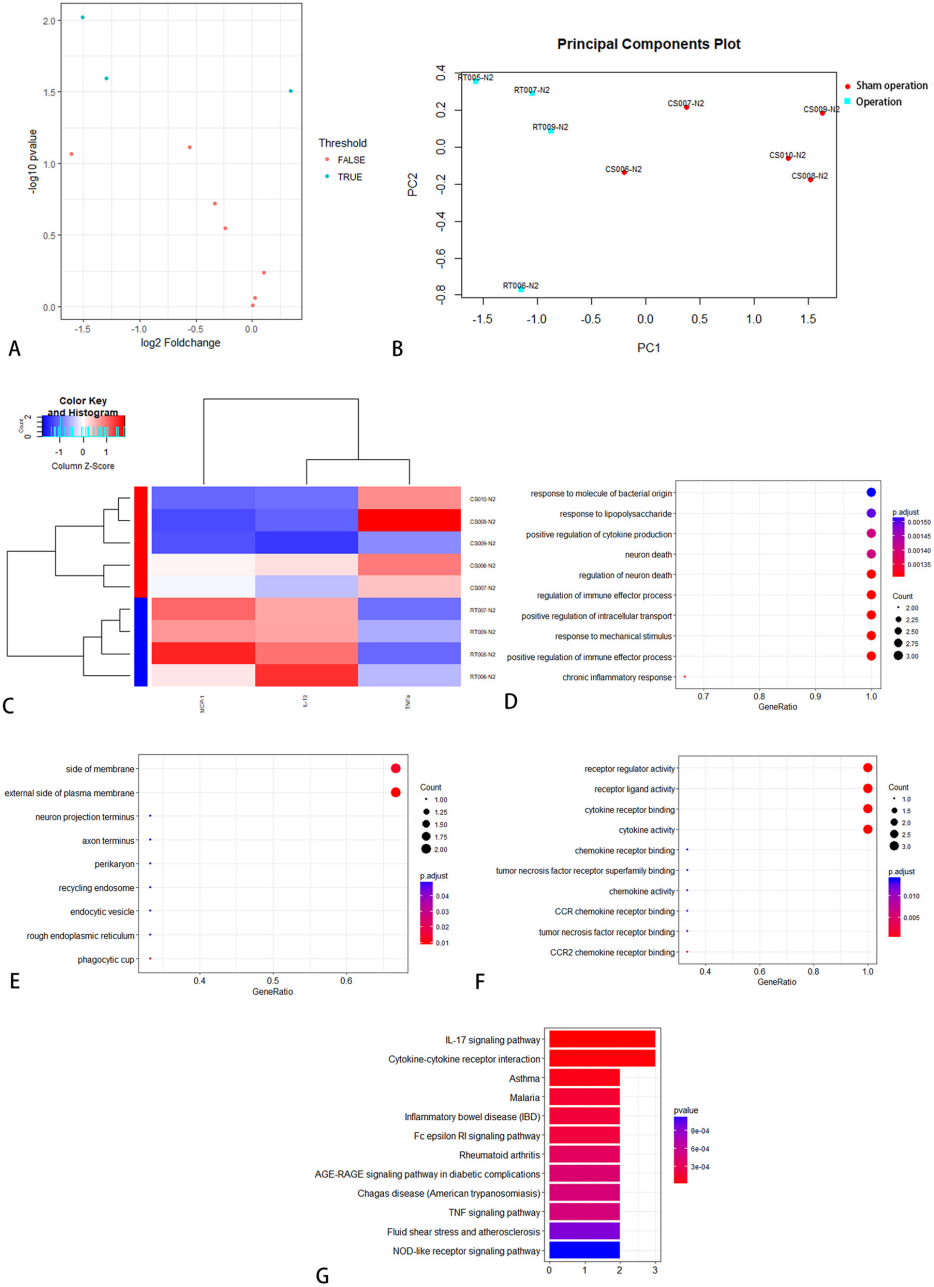
KEGG pathway analysis between operation group and sham operation group in SD rats on day 14.

On day 7, the expression levels of five cytokines (IL-2, IL-4, IL-10, IL-13, and TNF-α) were elevated in the sham-operated group compared to the control group, suggesting a potential association with the acute inflammatory response to surgical trauma (Table 5). KEGG enrichment analysis identified 29 significantly enriched pathways, and the top 12 were visualized in Figure 6. On day 14, the expression levels of six cytokines (IFN-γ, IL-1α, IL-2, IL-4, IL-13, and TNF-α) were elevated in the sham-operated group compared to the control group, suggesting a possible association with the chronic inflammatory response induced by surgical trauma (Table 6). KEGG enrichment analysis identified 43 significantly enriched pathways, and the top 12 were presented in Figure 7. Notably, IL-13 expression was higher on day 14 than on day 7 in the operated group (Figure 8). On day 7, three cytokines (IL-6, IL-13, and MCP-1) a differential expression pattern was observed between the operation group and the sham-operated group. Notably, IL-6 and MCP-1 were exhibited upregulation in the operation group, whereas IL-13 was showed a higher expression level in the sham-operated group (Table 7). KEGG enrichment analysis identified 20 significantly enriched pathways, with the top 12 pathways presented in Figure 9. On day 14, three cytokines, including TNF-α, IL-13, and MCP-1 were differentially expressed between the operated and sham-operated groups. Specifically, IL-13 and MCP-1 levels were elevated in the operated group, while TNF-α was more highly expressed in the sham-operated group (Table 8). KEGG pathway enrichment analysis uncovered 16 statistically significant pathways. Figure 10 provides a visual illustration of the top 12 significantly enriched pathways.

**Table 5:** Differentially expressed proteins in nerve tissues of sham operation group and blank group after 7-day injury of inferior brachial plexus in SD rats.

Factors	log2FC	P value
IL-2	0.588438	0.001469
IL-4	0.40449	0.004865
IL-10	0.56402	0.023297
IL-13	2.356023	7.45E-05
TNF-α	0.299466	0.034076

**Table 6:** Differentially expressed proteins in nerve tissues of sham operation group and blank group after 14 days of inferior brachial plexus injury in SD rats.

Factors	log2FC	P value
IFNg	0.671649	0.0040
IL-1a	0.584075	0.0058
IL-2	0.66235	0.0005
IL-4	0.376217	0.0297
IL-13	1.808767	0.0264
TNFa	0.482993	0.0094

**Table 7:** Differentially expressed proteins in nerve tissues of sham operation group and operation group after 7-day injury of inferior brachial plexus in SD rats.

Factors	log2FC	P value
IL-6	−1.73657	0.0136
IL-13	1.040548	0.0008
MCP-1	−2.07784	0.0113

**Table 8:** Differentially expressed proteins in proximal nerve tissue of sham operation group and operation group after 14 days of inferior brachial plexus injury in SD rats.

Factors	log2FC	P value
IL-13	−1.51131	0.009577
MCP-1	−1.29608	0.025317
TNFa	0.344974	0.031143

## 4 Discussion

In this study, we carried out an integrative analysis that combined clinical serum data and rat BPI models to explore the inflammatory mediators and neuroimmune pathways implicated in pain development subsequent to BPI. Our results unveiled distinct cytokine profiles in human serum and injured nerve tissues, identified key inflammation-related pathways, and demonstrated pathological and behavioral changes in line with neuroimmune activation.

Inflammatory cytokines and mediators including IL-18, CNTF, GM-CSF, IL-6, IL-13, and MCP-1 were significantly differentially expressed in the serum of BPI patients and the nerve tissues of BPI rats compared to healthy controls. These cytokines have previously been implicated in pain sensitization and peripheral immune activation. IL-1 family, IL-6 and TNF-α are well-established molecules in the inflammatory theory of neuropathic pain [17], [18], [19]. The IL-6/JAK2/STAT3 signaling pathway contributes to neuropathic pain development by modulating astrocyte and microglial activation following spinal cord injury [20]. Similarly, in a rat model of brachial plexus root avulsion, increased IL-1β, IL-6, and TNF-α expression is observed and is significantly suppressed by Acetylglutamine treatment [21], highlighting their potential as therapeutic targets. MCP-1 (CCL2) functions as a chemokine that recruits monocytes and other immune cells to injured sites and has been widely implicated as a driver of neuroinflammation [22]. In our study, MCP-1 expression was consistently elevated in the operated group, particularly in nerve tissues on days 7 and 14, as well as in the clinical pain subgroup. These findings were consistent with prior reports of increased MCP-1 levels in models of chemotherapy-induced neuropathic pain and chronic spinal cord compression [23], 24]. IL-18, a member of the IL-1 family, was notably upregulated in our clinical serum analysis of painful BPI patients. Previous studies have localized IL-18 expression to microglia, where it participates in microglia–astrocyte crosstalk through the IL-18/IL-18R axis, promoting the persistence of neuropathic pain [25]. In this study, we also found that IL-18 expression was significantly different between the pain and non-pain groups in BPI, suggesting that IL-18 may be involved in the progression of BPI-associated neuropathic pain. Moreover, Su et al. reported that BPI induces robust expression of IL-1β, IL-6, MCP-1, and Fizz1. Among them, IL-1β and IL-6 levels reach their peaks at the two – week mark, MCP-1 exhibits a transient increase, and Fizz1 increases at two weeks and returns to the baseline level by four weeks, suggesting a dynamic inflammatory and macrophage response [26]. In this study, significant differences in IL-6, IL-13, and MCP-1 were detected on day 7, while TNF-α, IL-13, and MCP-1 differences were observed on day 14 between surgical and sham groups, which indicated dynamic cytokine changes following BPI. We established a rat model of BPI with distinct injury subtypes (upper trunk, lower trunk, and complete avulsion) and examined both serum and nerve tissue responses at acute (day 7) and chronic (day 14) stages. While serum cytokine levels did not show significant differences in rats, inflammatory markers in nerve tissues, including IL-6, IL-13, MCP-1, and IFN-γ, were significantly elevated in the operated groups. Histological analyses revealed progressive structural disorganization and Schwann cell loss across injury subtypes, most pronounced in the complete avulsion group. These results highlight the importance of localized inflammatory responses at the injury site in shaping the pain phenotype. The observation that cytokines such as IL-13 and MCP-1 were elevated not only in operated but also in sham-operated animals suggests that surgical trauma itself can trigger measurable immune responses, which may confound interpretations in nerve injury models. Although our clinical analysis revealed differential expression of several inflammatory cytokines in the serum of BPI patients, no statistically significant changes were observed in SD rats after BPI induction. This discrepancy may reflect species-specific differences in immune response patterns. In human patients, inflammatory responses tend to be prolonged or fluctuate over time, owing to individual variability, comorbidities, and complex systemic regulation. In contrast, experimental animals, particularly SPF rats, exhibit more uniform and acute responses that may not fully replicate the human condition. Additionally, the selected timepoints for serum collection in rats (days 7 and 14 post-injury) may not coincide with the dynamic inflammatory process seen in patients, potentially resulting in oversight of transient cytokine changes. There were signal pathways related to autoimmune diseases identified, such as IL-17 signaling pathway, inflammatory bowel disease, rheumatoid arthritis, asthma, intestinal immune network produced by IgA, Th1 and Th2 cell differentiation. These findings imply that the development and persistence of pain after brachial plexus injury may not only be associated with peripheral and central inflammatory responses, but also potentially involve autoimmune mechanisms. In addition to well-characterized molecular targets, neuroimmune-related regulatory signaling pathways represent promising directions for future research. Following nerve injury, not only does local inflammation contribute to neuropathic pain, but neuroimmune interactions also play a pivotal role, particularly in BPI. IL-17 receptors are expressed in various neural tissues, where IL-17 can act directly on neurons or indirectly through satellite glial cells and immune cells to influence neural activity. For instance, IL-17 promotes oligodendrocyte proliferation and stimulates the secretion of pro-inflammatory cytokines [27]. In multiple sclerosis models, IL-17 has been shown to activate the p38 signal pathway, exacerbating inflammation and inducing demyelination of nerve fibers; conversely, IL-17 gene knockout significantly mitigates these pathological processes [28]. IL-17 is implicated in the neuropathogenesis of multiple sclerosis, epilepsy, and ischemic brain injury. In the dorsal root ganglion and spinal cord, IL-17A enhances nociceptive transmission in both neuropathic and inflammatory pain. Moreover, IL-17A can stimulate microglia to secrete neurotrophic factors such as NGF, BDNF, and GDNF. Therefore, elucidating the upstream regulators of IL-17 signaling and its downstream neuroimmune networks may provide new insights into the mechanisms of neuropathic pain after brachial plexus injury. Serum, cerebrospinal fluid, and nerve tissue are all feasible sample sources for protein microarray screening. In this study, serum samples from patients were chosen for the following reasons: (1) serum collection is straightforward, convenient, and minimally invasive; (2) CSF acquisition is more invasive and clinically challenging; (3) brachial plexus stump tissues are scarce, and dorsal root ganglia, typically accessible only during intraoperative exploration, are limited and highly valuable; and (4) following brachial plexus root avulsion injury, the integrity of the blood–brain barrier is compromised, allowing serum cytokine profiles to partially mirror central and peripheral immune responses. Although serum samples may not provide as direct evidence as nerve tissues, our findings suggest that they can effectively reflect alterations in inflammatory factor expression after nerve injury. These results offer a systemic perspective and may offer insights future strategies for the diagnosis and treatment of brachial plexus injury.

This study also has certain limitations. First, the sample size of patients with BPI was relatively small. Second, the generalizability of our findings may be constrained by  the inclusion of patients from a single hospital, which may not fully represent the broader BPI population. Third, due to ethical considerations and the invasiveness of nerve biopsy in BPI patients, we failed to obtain nerve tissue specimens from the clinical cohort. Finally, while the study identified differentially expressed proteins and associated pathways, the precise mechanistic links between these factors and the processes of pain or injury in BPI remain elusive. These mechanisms will be further explored in our future research. In conclusion, our findings further support the inflammatory hypothesis of neuropathic pain. Cytokines appear to contribute to the development of neuropathic pain not only through classical inflammatory pathways but also via neuroimmune mechanisms, such as the IL-17 signaling pathway. The cascade initiated by IL-18 as a proinflammatory cytokine, as well as T cell-mediated activation of the IL-17 pathway, warrants further investigation as a potential mechanism underlying neuropathic pain following brachial plexus root avulsion injury.

## Supplementary Material

Supplementary Material

Supplementary Material

Supplementary Material

Supplementary Material
