# Supplementary figures and images for: Neuroimmune related pathway may involve in neuropathic pain after brachial plexus injury: a clinical and experimental discovery
Source: Open Life Sci. 2026 May 11;21(1):20262001. doi: 10.1515/biol-2026-2001 (PMC13157261; doi:10.1515/biol-2026-2001)

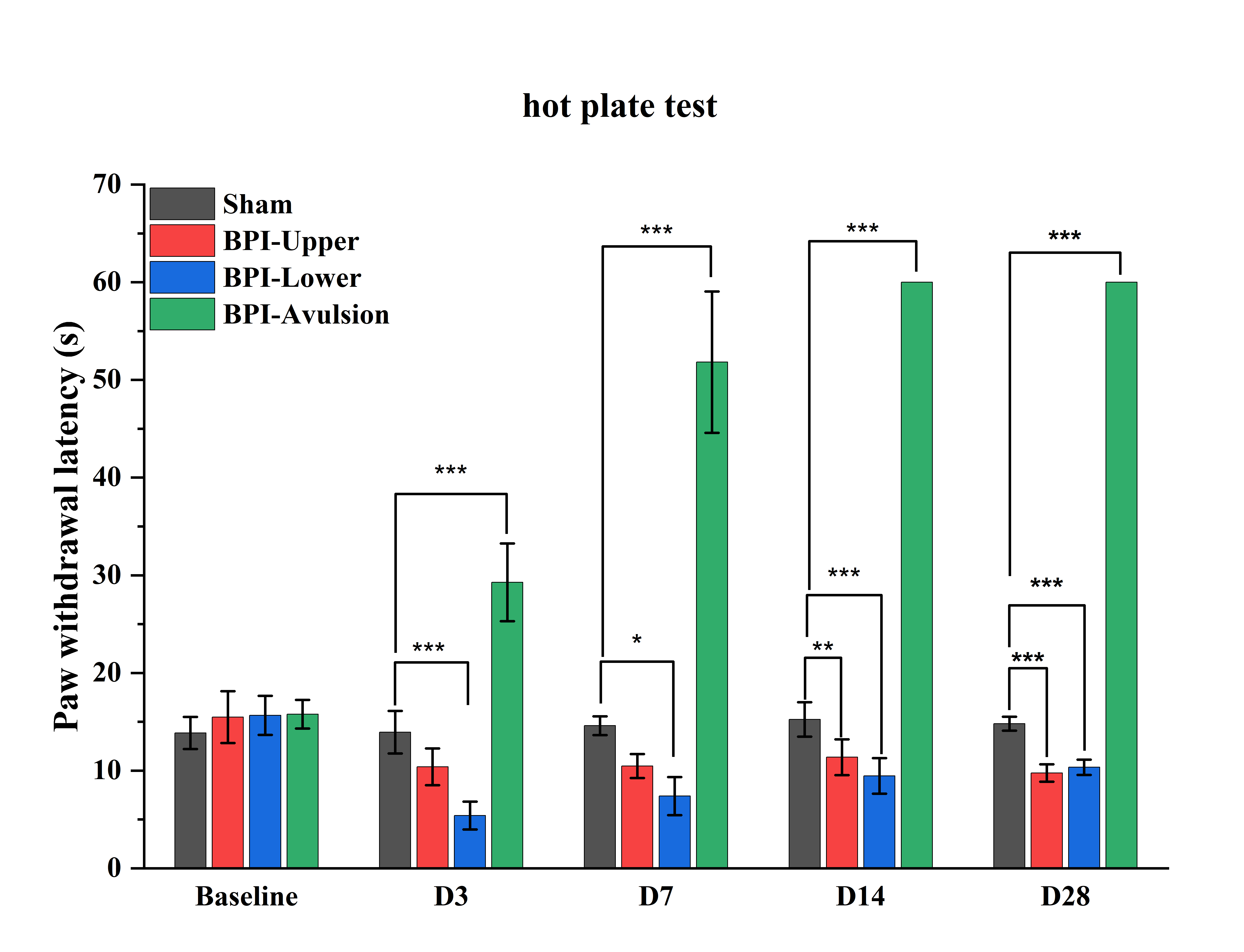

Supplement: Supplementary file 1 — Supplementary Material [file j_biol-2026-2001_suppl_001.png]

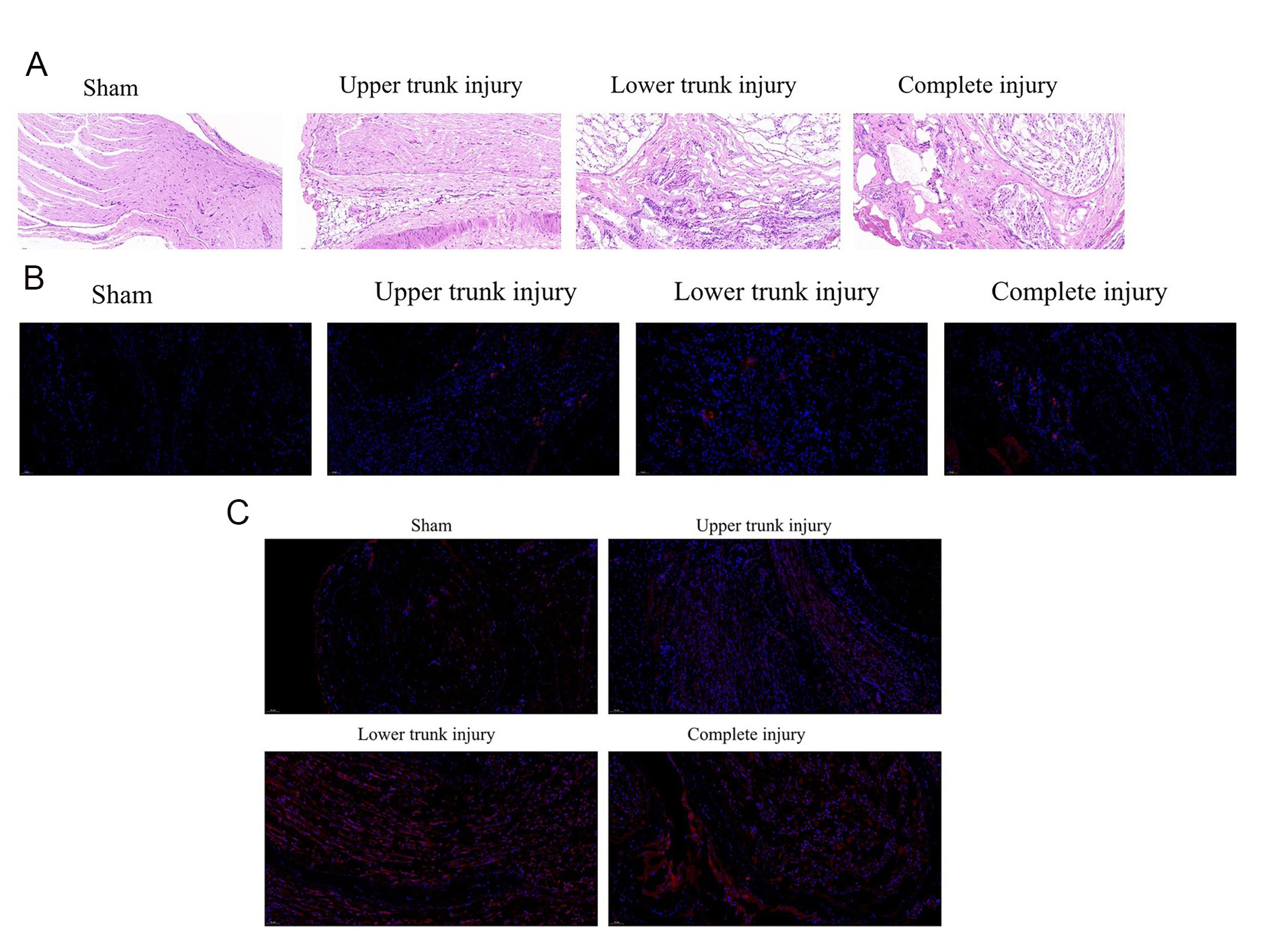

Supplement: Supplementary file 2 — Supplementary Material [file j_biol-2026-2001_suppl_002.zip › j_biol-2026-2001_suppl_002.tif]

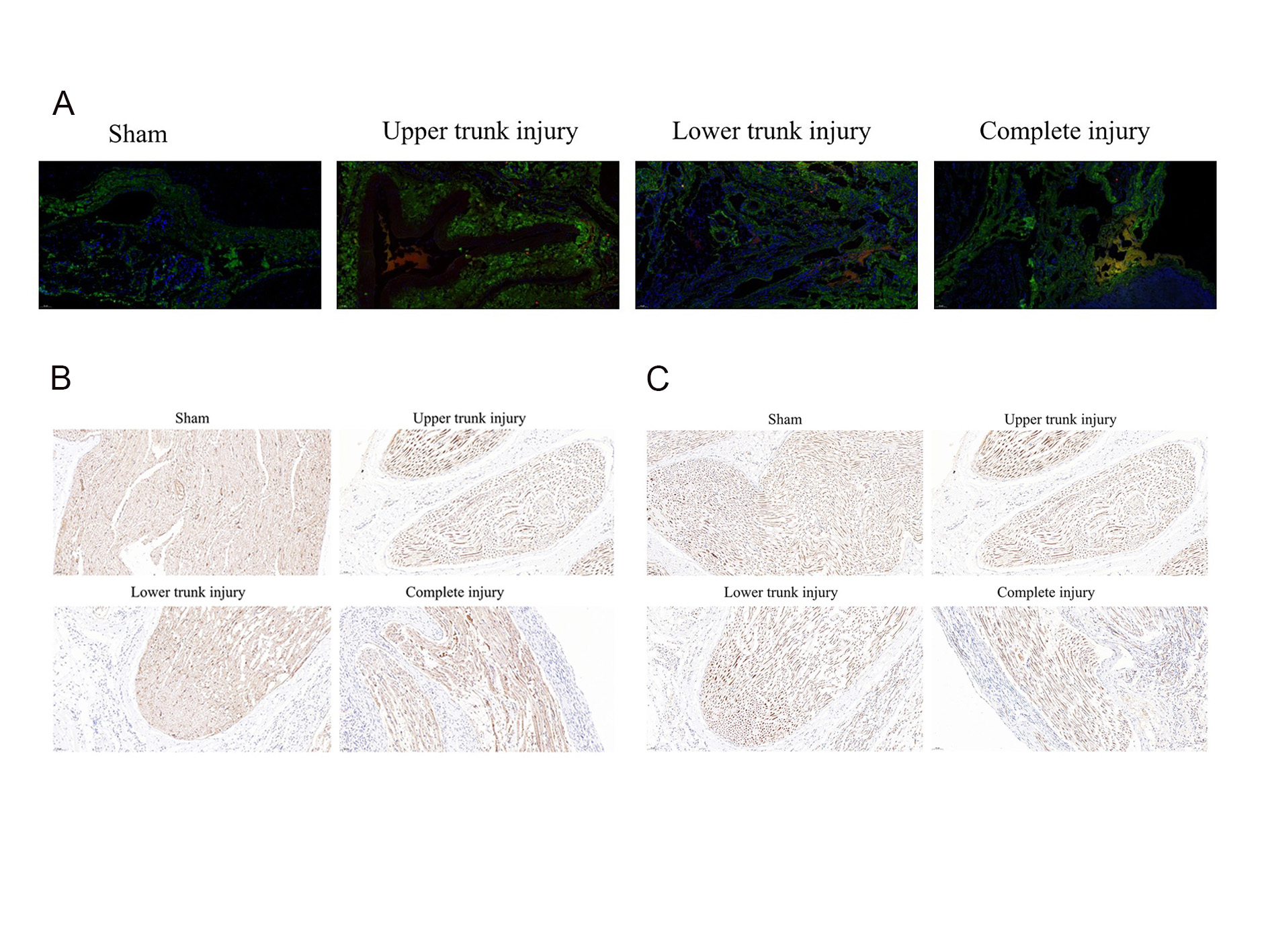

Supplement: Supplementary file 3 — Supplementary Material [file j_biol-2026-2001_suppl_003.zip › j_biol-2026-2001_suppl_003.tif]
