# Supplementary material for: Neuroimmune related pathway may involve in neuropathic pain after brachial plexus injury: a clinical and experimental discovery
Source: Open Life Sci. 2026 May 11;21(1):20262001. doi: 10.1515/biol-2026-2001 (PMC13157261; doi:10.1515/biol-2026-2001)
Supplement: Supplementary file 4 — Supplementary Material [file j_biol-2026-2001_suppl_004.docx]

Supplementary Figure 1 Hot plate test assessing thermal nociception following different types of BPI. Paw withdrawal latency was measured at baseline and on postoperative days 3, 7, 14, and 28 in four groups: sham, BPI-upper, BPI-lower, and BPI-avulsion. Data are presented as mean ± SD. Statistical comparisons were performed using ANOVA followed by Tukey's test. ^*^P < 0.05, ^**^P < 0.01, ^***^P < 0.001.

Supplementary Figure 2 Histological and immunofluorescence assessment of nerve injury in different types of BPI. (A) Representative hematoxylin and eosin (HE)-stained sections of proximal nerve tissues of the brachial plexus obtained from sham-operated rats and rats with upper trunk injury, lower trunk injury, or complete BPI. (B) Immunofluorescence staining of TrkA. Frozen proximal nerve tissues of the brachial plexus sections were stained with anti-TrkA primary antibody followed by Alexa Fluor-conjugated secondary antibody (red). Nuclei were counterstained with DAPI (blue). (C) Immunofluorescence staining was performed to assess TRPV1 distribution using anti-TRPV1 (red) and DAPI (blue), with fluorescence imaging used to compare expression across different injury groups. Scale: 50 μm; Magnification: 200×.

Supplementary Figure 3. Histological and immunohistochemical analysis of neuronal and Schwann cell markers following different types of BPI. (A) Representative immunofluorescence images showing expression of choline acetyltransferase (CHAT, green) and calcitonin gene-related peptide (CGRP, red) in the proximal nerve tissues of the brachial plexus among sham, upper trunk injury, lower trunk injury, and complete injury groups. Nuclei were counterstained with DAPI (blue). (B) Immunohistochemical staining of S100, a Schwann cell marker, was performed to evaluate peripheral nerve regeneration across different injury groups. (C) Immunohistochemical staining of NF200, a neurofilament marker for neuronal cell bodies and axons, was used to assess nerve fiber integrity. Scale: 50 μm; Magnification: 200×.
